# Sealing ability in vitro study and biocompatibility in vivo animal study of different bioceramic based sealers

**DOI:** 10.1002/cre2.652

**Published:** 2022-11-17

**Authors:** Tara H. Haji, Bahar J. Selivany, Abdulhaq A. Suliman

**Affiliations:** ^1^ Kurdistan Board for Medical Specialties Duhok Iraq; ^2^ College of Dentistry University of Duhok Duhok Iraq; ^3^ College of Dentistry Ajman University Ajman United Arab Emirates; ^4^ Centre of Medical and Bio‐allied Health Sciences Research Ajman University Ajman United Arab Emirates

**Keywords:** bio_ceramic, biocompatibility, sealing ability, SEM

## Abstract

**Introduction:**

The effectiveness of root canal therapy in endodontic practice is largely determined by providing a compact fluid‐tight closure at the apex of the root canal, which inhibits irritant entry and buildup, which leads to a biological breakdown of the attachment mechanism and failure. During obturation, along with gutta‐percha, root canal sealers are employed to fill voids and seal root canals. Root canal sealers come in a variety of shapes and sizes, each with its own set.

**Aim:**

Evaluation of sealing ability in vitro study by using scanning electron microscopy (SEM) and biocompatibility in vivo animals study of BioRoot RCS and meta Biomed bio_ceramic sealer (CeraSeal RCS) and compared the findings with that of Zinc oxide eugenol (ZOE) sealer as control.

**Materials and Methods:**

This study utilized two bio_ceramic sealers (BioRoot RCS and meta Biomed bio_ceramic sealer (CeraSeal RCS) and compared the findings with that of ZOE sealer as control. Biocompatibility was determined by examining histopathological biopsy specimens collected from rabbits. Each rabbit had four dentin tubes implanted into the subcutaneous tissues, one for BioRoot RCS, one for CeraSeal RCS, and one for ZOE RCS, with the fourth tube being empty haematoxylin and eosin were used to stain histological sections, and a light microscope was used to evaluate them. Extracted human single canal premolars were used to evaluate the sealing ability. The root canals were divided into three sections (coronal, middle, and apical). SEM was used to assess the adhesion quality at the sealer‐dentin interface.

**Results:**

BioRoot and CeraSeal sealers have excellent sealing adaptation and biocompatibility, as well as rapid tissue recovery, while ZOE sealers have a slower recovery of inflammatory reaction results when compared to bio_root and ceraSeal sealers, as well as a less sealing adaptation than the two other bio_ceramic sealers.

**Conclusion:**

In general, the two bioceramic sealers tested were biocompatible and capable of sealing or adhesion. While ZOE had less adherence ability and less biocompatibility.

## 1 INTRODUCTION

Endodontic treatment is a set of procedures used to treat an infected canal in a tooth, with the aim of ending the infectious process and preventing new microbial (Hargreaves & Berman, 2016). The use of root canal sealers to execute root canal filling in obturation procedures is essential in preventing treatment failure (Johnson et al., 2016). As a result, these materials should contain a set of properties that permit successful endodontic filling. The most significant characteristics are (a) having a fluid‐tight seal, (b) antibacterial activity (or at least not encouraging bacterial development), (c) being biocompatible and not irritating to radicular tissue, and (d) not staining tooth structure (Grossman, 1988). Scientific and technological advancements have allowed materials to improve equipment and materials in a range of fields over time (Kishen et al., 2016), particularly in endodontics, thus providing superior physical results like sealing ability to the root canal dentin (Grossman, 1988). Notably in endodontics, therefore delivering superior physical effects such as root canal dentin sealing ability (Grossman, 1988). The ability to seal is determined by the material's resistance to microleakage through the thickness (Al‐Haddad et al., 2015; Hovland & Dumsha, 1985), and the long‐term success of endodontic therapy is determined by full filling after root canal obturation (Camilleri et al., 2011). Because of poor contact in the middle of the gutta‐percha and the sealer, as well as the dentin, microleakage is one of the important sources of endodontic failure (Drukteinis et al., 2009; Nair et al., 2011; Trope et al., 1995). Stereomicroscopy, scanning electron microscopy (SEM), and leakage tests have all been used to assess a sealant's adaptability to dentin (Gomes‐Filho et al., 2012; Peters et al., 2000). The specific aim of this study is to evaluate the sealing ability and biocompatibility of BioRoot RCS and meta Biomed bio_ceramic sealer (CeraSeal RCS) and compare the findings with that of Zinc oxide eugenol (ZOE) sealer as control. The sealers that were used in this study were chosen because there is no previous study comparing the sealing ability and biocompatibility between tricalcium silicate sealers (bioroot, ceraseal sealers) and resin base sealers. In this investigation, SEM will be used to assess the sealing ability. Sealers' adhesion ability is scored as either very good, good, or acceptable (Ray & Seltzer, 1991). There have been several materials developed, and they can be categorized into the following classes based on their chemical composition and structure: Bioactive endodontic sealers, resin‐based endodontic sealers, silicone‐based endodontic sealers, calcium hydroxide‐based endodontic sealers (Gambarini et al., 2021; Gomes‐Filho et al., 2012; Khandelwal & Ballal, 2016). CeraSeal (CS) (MetaBiomed Co.) is an antibacterial calcium‐silicate‐based premixed substance that never shrinks and has a high pH level (López‐García et al., 2020). There is also a BioRoot BC sealer (Septodont) that is a tricalcium silicate bioactive sealer that comes in powder and liquid form (Kharouf et al., 2020). It possesses excellent characteristics (Jain & Ranjan, 2015; Santos et al., 2019). Along with the ZOE sealer, these two bioceramic sealers will be used in this investigation. A study by Hanseul Oh and Egan Kim evaluated the biocompatibility of calcium silicate‐based sealers (CeraSeal and EndoSeal TCS) and epoxy resin‐based sealers (AH‐Plus) in terms of cell viability, inflammatory response, expression of mesenchymal phenotype; they found a low degree of cell proliferation on AH‐Plus and a high degree of cell proliferation on calcium silicate‐based sealers. In this study, calcium silicate‐based sealers appear to be more biocompatible and less cytotoxic than epoxy resin‐based sealers low degree of cell proliferation on AH‐Plus, and a high degree of cell proliferation on calcium silicate‐based sealers. Biocompatibility is the most important feature of root canal sealers since they come into contact with periradicular tissues (Al‐Haddad et al., 2015). This biocompatibility refers to the ability to elicit an appropriate host response in a specific application; that is, it does not cause an adverse reaction when it comes into contact with the tissue, which can be determined by looking for cell infiltration or vascular changes, as well as determining the intensity of the inflammatory reaction (Ricucci & Langeland, 1998; Santos et al., 2019). The test sealers are implanted via surgery in these studies, and the body's response to tissue injury begins with inflammation and progresses via wound healing mechanisms, (Cosme‐Silva et al., 2019; Pinheiro et al., 2018; Simsek et al., 2015; Tyagi et al., 2013). If a normal healing process is followed, the implanted substance can be considered biocompatible (Cosme‐Silva et al., 2019; Ricucci & Langeland, 1998). In certain investigations, the materials to be examined may be directly injected subcutaneously, while in others, the materials should be implanted in tubes, such as a dentin tube or a polyethylene tube (Bernath & Szabo, 2003; El‐Mansy et al., 2020).

## 2 MATERIAL AND METHODS

### 2.1 Biocompatibility test

Thirty healthy local male rabbits weighing 350 ± 50 g from the local area were used. This study was approved by the Local Ethics Committee at the College of Veterinary Medicine, University of Duhok. All animals were implanted with four dentin tubes. Each tube was prepared from roots derived from the palatal and distal roots of the maxillary 1st molars free from caries and crack. Protaper files and Gate Glidden burs from Dentsply Maillefer were used for the preparation of the dentin tube, resulting in an exterior wall thickness of 0.5–1 mm. The apical foramina were enlarged to an approximate diameter of 2 mm, and the length was 2 mm. the master apical file was F3. Each dentine tube was irrigated with 2 ml and 3% sodium hypochlorite for 30 min and 18% ethylene diamine tetraacetic acid (EDTA) for 2 min, then rinsed with distilled water and autoclaved. The pressure of approximately 15 pounds per square inch was used to achieve a chamber temperature of at least 250°F (121°C) for a prescribed time—usually 30–60 min (Holland et al., 1999).

Each dentin tube was disinfected in 2.2% glutaraldehyde for 12 h before surgery and sterilized in an autoclave. There were three groups of rabbits separated according to study time (96 h, 10, and 21 days), each of the 10 rabbits was implanted with four dentin tubes subcutaneously. The first tube was filled with Ceraseal, second tube with Bioroot, third tube with ZOE, and the last tube was left empty. The volume of sealers was applied in a standardized manner. Anesthesia was given to the animals at a rate of 0.001 mg/kg body weight (Santos et al., 2019). Ten percent of Ketamine Hydrochloride (Cheminova) was used to achieve this effect, and it was delivered intraperitoneally associated with Xylazine Hydrochloride (10 mg/kg). After shaving and disinfection of surgical sites, for placement of tubes in the anterior and posterior sections of the dorsum four roughly 4 mm incisions were made with a number 15 scalpel blade (Denti‐Lab). The tubes were longitudinally implanted in animals, with the first three tubes filled with root canal sealers and the fourth one left unfilled. A 6.0 nylon thread was used for suturing (Ethicon). Animals were euthanized with anesthetic overdose and slaughtered at 96 h, 10, and 21 days (Ketamine hydrochloride; Cheminova) was used. Excision biopsy of areas at the boundary of the implants was used to check the tissue reaction to implanted materials. Tubes were dislodged from sections without touching tissue extremities using an incision on the longitudinal axis. The samples were fixed in 10% formalin for 2 days. Specimens were placed in paraffin blocks. Serial sections of 4 µm thickness were developed by a rotary microtome for further staining with hematoxylin and eosin (H&E) stain.

A microscope at 400× and 100× magnification (Prior Scientific company) was used to evaluate the presence of inflammatory cells, such as polymorphonuclear neutrophils, lymphocytes, macrophages, eosinophils, and giant cells close to the tube opening, with edema and also granulation tissue formation. Fibrous capsule thickness was assessed close to the tube opening.

I—Inflammatory response scored as (Mori et al., 2014; Santos et al., 2019):

1: Few or absence (0_5);

2: Mild (5_25);

3: Moderate (25_125);

4: Severe(up to 125) inflammatory cells.

II—Edema scores:

0 = No edema;

1 = Mild edema;

2 = Moderate edema;

3 = Severe edema.

III—Granulation tissue:

0 = No granulation tissue;

1 = Early granulation tissue;

2 = Prominent granulation tissue.

For fibrous capsule, thickness scored either thin fibrous capsule when less than 150 μm and thick when more or equal to 150 μm (Mori et al., 2014; Santos et al., 2019).

The samples were examined by a histopathologist, and the data were collected and analyzed statistically.

### 2.2 Sealing ability test

Thirty comparable in size, single‐rooted human mandibular premolars were taken from patients in the hospital for this study, after obtaining verbal informed agreement for using these teeth in the research. The similarity size of teeth was evaluated by periapical x‐ray, they were free from caries. Within 2 h, 5.25% sodium hypochlorite was used to disinfect all the teeth. Until further testing, the teeth were kept in disinfectant solution(0.1%) thymol crystals. Preoperative radiographs in the mesiodistal and buccolingual directions were taken to establish the existence of a single root canal that is free of root caries, resorption, or calcification. The crowns of all teeth were removed at the cementoenamel junction, and each root was set to about 12 mm in length. A #10 K‐File (Dentsply Maillefer) was placed into the root canal until it reaches the apex. The working length was set by subtracting 0.5 mm from this length. Instrumentation of all teeth done to a size of 40/06 using a crown‐down approach by using protaper universal files system (Dentsply Tulsa Dental Specialties). Between each instrument, for 30 min irrigation of 2 ml of 2.5% NaOCl were utilized. To remove the smear layer, a final 1‐min rinse was conducted with 2 ml 2.5% NaOCl, 2 ml 17% EDTA (Patterson Dental Supply), and 10 ml distilled water as a final rinse. Root canal sealers were mixed according to the manufacturer's recommendations and placed into the canal using a size 40 lentulo spiral (Produits Dentaires SA) to distribute sealers equally throughout the canal and using the single cone technique for obturation. According to the following groups:

Group 1: 10 roots were filled using CeraSeal Root Canal Sealer with 40/06 gutta‐percha.

Group 2: 10 roots were filled using BioRoot canal Sealer with 40/06 gutta‐percha.

Group 3: 10 roots were filled using a ZOE canal sealer with 40/06 gutta‐percha (Shangahi company).

The roots were kept at 37°C and 100% humidity for 5 days after filling to permit the sealer to be fully set. Roots were sectioned horizontally in the labiolingual direction and separated into apical (0–4 mm), middle (4–8 mm), and coronal (8–12 mm) sections with a thickness of about 2 mm were then prepared to be examined using SEM Sections were vacuum dried, gold‐cover, and then inspected using a SEM (Carl Zeiss NTS GmbH). At a magnification of 2000×, the adaptation of each sealer to the dentin was evaluated from the coronal to apical ends, and microphotographs were taken. If a gap presents between the materials and dentin, it was measured by the J image software program.

Scores of sealing ability (Ray & Seltzer, 1991):

1. Very good adhesion; contact line on the sealer‐dentine interface with no gaps.
2. Good adhesion; with a curved contact line on the sealer‐dentine interface and minor gaps between sealer and dentine wall.
3. Acceptable adhesion; gaps were frequently discovered in the middle of the sealer and dentine wall, and the sealers‐dentine interface had an indistinct and very curved contact line.

## 3 RESULTS

### 3.1 Biocompatibility

The severity of the inflammatory response (inflammatory cell scores) in all experimental groups was studied histopathologically and statistically during the subcutaneous implantation period.

#### 3.1.1 Control group

**Figure A1:**
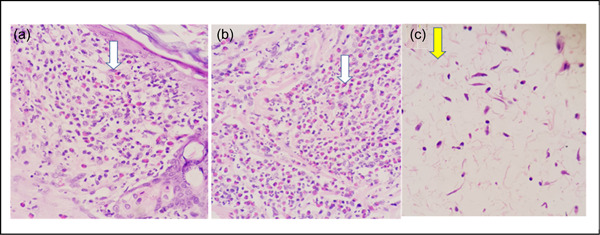
Control group: Dense eosinophil aggregates throughout the wall (white arrows) indicating allergic reaction (H&E, (a) ×400, (b) ×400); mild inflammation showing edema (yellow arrow) within inflammatory infiltrates (H&E, (c) ×400).

At 96 h, a severe reaction was noted, the tissue was invaded with Dense eosinophil aggregates throughout the wall indicating an allergic reaction as shown in Figure A1a,b. At Day 10, the severity of the reaction was moderate to mild and the tissue was marked with Some areas of congested blood vessels. At Day 21 (Figure A1c), mild inflammation showed small edema (yellow arrow) within inflammatory infiltrates.

#### 3.1.2 CeraSeal RCS

**Figure A2:**
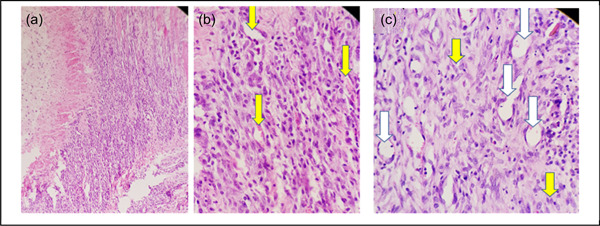
CeraSeal RC: Severe acute inflammation with the beginning of granulation tissue (yellow arrow) within inflammatory infiltrates (H&E, (a) ×100, (b) ×400); mild inflammation showing edema (yellow arrow) within inflammatory infiltrates (H&E, (c) ×400).

At 96 hrs (Figure A2a,b), a severe inflammatory reaction was noted, with the beginning of granulation tissue. The severity of the inflammatory reaction was moderate to mild on the 10th day as shown in. On the 21 Granulation tissue formation showing proliferating blood vessels (white arrows) and fibroblasts (yellow arrows), as shown in Figure A2c.

#### 3.1.3 Zinc oxide eugenol RCS

**Figure A3:**
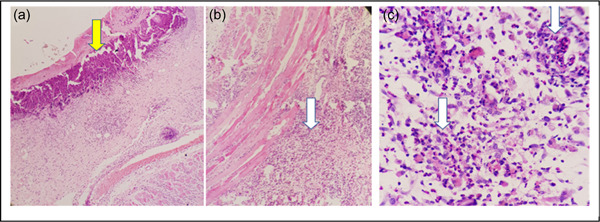
Ulceration (yellow arrow) with suppurative inflammation throughout the wall (white arrows) (H&E, (a) ×100, (b) ×100, (c) ×400).

At 96 h (Figure A3a) ulceration was noted with severe suppurative inflammation throughout the wall was seen in (Figure A3b,c). At Day 10, the inflammatory reaction was still severe. At Day 21, dilated blood vessels were detected with a considerable number of chronic inflammatory cells.

#### 3.1.4 BioRoot RCS

**Figure A4:**
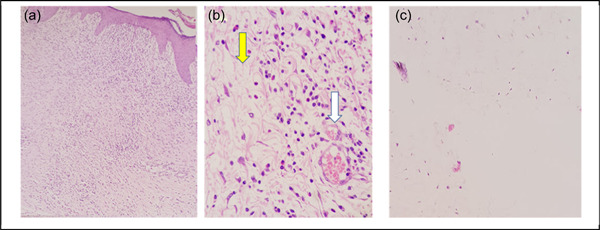
BioRoot sealer: Moderate acute inflammation showing edema (yellow arrow) within inflammatory infiltrates with some congested blood vessels (white arrow) (H&E, (a) ×100, (b) ×400); milder to the absence of inflammation with blood vessels formation (H&E, (c) ×400).

At 96 h, a severe to moderate inflammatory reaction was noted. On Day 10, the inflammatory reaction was moderate with edema, and few dilated blood vessels were observed, as shown in Figure A4a,b, with small dilated blood vessels. At Day 21, a milder inflammatory reaction with blood vessel formation was seen, as shown in Figure A4c.

#### 3.1.5 Statistical analysis

The mean values and standard deviation were estimated using the obtained data; finding the statistically significant difference between the three groups studied using SPSS version 20 (Table A1 in Appendix) displays the sample numbers in each period and each group, and there was a statistically significant difference between ZOE and CeraSeal, BioRoot with *p* .05 using analysis of variance (ANOVA) test and least significant difference (LSD) test which mean is the value at a particular level of statistical probability (e.g., *p* ≤ .01 means with 99% accuracy) when exceeded by the difference between two varietal means for a particular characteristic, then the two varieties are said to be distinct for that characteristic at that or lesser in Table A1 at 2nd and 3rd study period. At 96 h, according to a histological investigation, all of the sealers examined had substantial inflammatory cell infiltration. There was no variance between the groups (*p* .647). BioRoot RCS exhibited quick recovery, and moderate to mild inflammatory response at Day 10, which was equivalent to control. CeraSeal RCS also showed good recovery, but ZOE RCS exhibited greater inflammation than control and the other two experimental root canal sealers, between the study periods, there were statistically significant differences (*p* = .000). At Day 21, over time, the inflammatory response considerably subsided for every tested group at (*p* = .000). A significant difference was noted in Zinc oxide when compared to control, BioRoot, and CeraSeal RCS.

### 3.2 Sealing ability

#### 3.2.1 CeraSeal RCS

**Figure A5:**
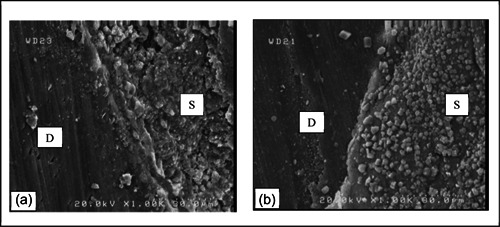
SEM images of CeraSeal RCS (a), BioRoot(b) at magnification 2000×. SEM, scanning electron microscopy.

The interface between CeraSeal RCS with the root canal dentin wall (D) showed very good sealing ability in all parts of the root canal (coronally, middle, and apically) with sealer (S), as shown in Figure A5a.

#### 3.2.2 BioRoot RCS

The interface between bio_root sealer with the root canal dentin wall showed very good sealing ability in all parts of the root canal (coronal, middle, and apical) through SEM image analysis at 2000× magnification, as shown in Figure A5b.

#### 3.2.3 Zinc oxide eugenol RCS

**Figure A6:**
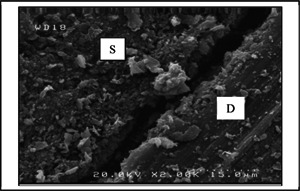
SEM images of Zinc oxide eugenol. RCS at magnification 2000×. SEM, scanning electron microscopy.

The interface between ZOE with the root canal dentin wall (D) showed acceptable adhesion in the presence of a gap in all parts of the root canals (coronal, middle, and apical), as shown in Figure A6.

#### 3.2.4 Statistical analysis

The mean values and standard deviation were estimated from the obtained results; find the significant difference between the three groups studied using SPSS version 20 provided in the appendix (Table A2). There was a statistically significant difference noted between ZOE, CeraSeal, and BioRoot RCS with *p* ≤ .05 using ANOVA test and LSD test in Table A2. LSD test mean is the value at a particular level of statistical probability (e.g., *p* ≤ .01 means with 99% accuracy) when exceeded by the difference between two varietal means for a particular characteristic, then the two varieties are said to be distinct for that characteristic at that or lesser. Root canals sealed with tested root canal sealers showed through SEM analysis that their contact with the dentin was sufficient throughout the root canal. The sealing capacity was improved in the BioRoot RCS and CeraSeal RSC, while ZOE RCS had a gap between the dentin wall and the gutta‐percha.

## 4 DISCUSSION

In order to achieve successful endodontic treatment and a good prognosis, it is crucial to have a tight apical seal which is dependent on proper instrumentation and cleaning of the root canal system in conjunction with adequate obturation (Al‐Haddad et al., 2015). Penetration of root canal sealers into dentinal tubules provides a better sealing ability, thus preventing residual bacteria from re‐growing within the tubular space (Camilleri et al., 2011). Calcium silicate‐based sealers have an alkaline pH, are hydrophilic by nature, insoluble in tissue fluids, and do not shrink when they are set. Additionally, the moisture environment of the tooth affects the root canal dentin and bioceramic sealers adhesion (Camps et al., 2015). These sealers are characterized by their potential bioactive properties, where calcium hydroxide and hydroxyapatite are formed once the sealer contacts water, resulting in a high alkaline pH that activates and initiates the expression of alkaline phosphatase, favoring the formation of mineralized tissue and possessing an antibacterial effect. Several methods have been postulated for evaluating the sealing ability: dye penetration, fluid filtration techniques, radioisotopes, SEM analysis, electrochemical leakage tests, glucose penetration, and bacterial penetration test (Xuereb et al., 2015).

SEM was utilized in this study as it allows proper evaluation of the sealing ability and adhesiveness of the sealer to dentin walls or sealer‐gutta‐percha interface on the various levels of root sections (Gomes‐Filho et al., 2012). Moreover, it provides high magnification, allowing better observation of surface topography (Peters et al., 2000). Therefore, this study aimed to evaluate and compare the sealing ability of two types of calcium silicate‐based sealers and a ZOE root canal sealer using SEM.

Results of the current study revealed that groups A and B had higher adhesion strength than group C in the three root sections, namely coronal, middle, and apical. This can be attributed to the excellent physical properties of the calcium silicate‐based sealers, such as flow, low film thickness, and dimensional stability (Kharouf et al., 2020). In addition to the alkaline nature of the byproducts produced by the calcium silicate‐based sealers that might have denaturized the dentin collagen fibers thus facilitated the sealers penetration (Xuereb et al., 2015).

While, the lower adhesive strength exhibited by group C could be due to the incomplete polymerization and the setting shrinkage of its resinous components resulting in the formation of poor microtags which consequently exhibits low adhesion properties (Tyagi et al., 2013).

The fact that degree of adhesion of the sealers to the dentin wall depends on the surface energy of the dentin, surface tension, and wettability of the sealer in addition to the cleanliness of the dentin surface (Nair et al., 2011). Dentin in the coronal, middle, and apical sections has different surface energies, in conjunction with obstacles faced during complete removal of the smear layer from the apical region might be the cause of its lower sealer penetration (Nair et al., 2011).

These results were confirmed by the descriptive characteristics of the absence of gaps revealed by the SEM study as shown in Figure A5. Where groups A and B demonstrated a clearly recognizable adhesion without gap presence, However, group C showed Gap percentages. The images obtained through scanning electron microscopy have some limitations; are only representative of sectioned canal levels examined. Further studies are required to evaluate the mineralogical characteristics of both BioRoot and CeraSeal when it is in contact with different solutions, in addition to their physiochemical properties.

The second purpose of this study was to compare the biocompatibility of the BioRoot RCS and CeraSeal RCS in vivo study to ZOE RCS. The evaluation of biocompatibility of any dental material intended for clinical use necessitate a planned assessment in sequential stages, including in‐vitro cell line cultures, tissue reaction in animals, and clinical trials, to protect the patient from possible hazards (Ricucci & Langeland, 1998; Santos et al., 2019).

Three observation time intervals were used to examine the intensity of the inflammatory reaction of the tested materials. At first observation time (96 h), histological analysis revealed that all tested sealers showed severe inflammatory cell infiltration in a thick poorly organized fibrous capsule, a significant difference was not noted between the groups (*p* > .05). The tissue was invaded with dense eosinophil aggregates throughout the wall indicating allergic reaction as shown in Figure A1a,b. The cause of these histological findings were due to surgical trauma of the incision and the physical presence of the tubes may be caused by the initial inflammatory reaction in the control group (Cosme‐Silva et al., 2019). A strong inflammatory reaction was seen in both two calcium silicate sealers. Materials made of calcium silicate are known to release calcium ions when they come into contact with tissue fluids. Thus, the rising alkaline pH after setting could be responsible for the initially significant inflammatory response. Additionally, the heat produced during the setting process encourages the recruitment of inflammatory cells, which releases cytokines. Pinheiro et al. (2018). However, the inflammation reduced rapidly. At Day 10, BioRoot RCS showed rapid recovery and mild inflammatory to a moderate response similar to control. CeraSeal RCS also showed rapid recovery and mild inflammation to the moderate response, while ZOE RCS still exhibited greater inflammation than controls. When the ZOE sealer comes into contact with the tissue, it generates a strong inflammatory reaction (Simsek et al., 2015). There were statistically significant differences (0.000) between the study groups as shown in Table A1. In‐vitro study on BioRoot by Camps et al. (2015) showed that calcium hydroxide released during setting caused mild to moderate inflammatory reactions in viable cells. As shown in histological analysis at second period of observation of both BioRoot and CeraSeal RCS.

A well‐defined fibrous capsule with granulation tissue formation that containsproliferating blood vessels and fibroblasts, as shown in Figure A2c, was found in both calcium silicate sealers at the end of 21 days which indicates favorable interaction with the adjacent living tissues. Thus, the biocompatibility of BioRoot and CerSeal RCS may be related to the Ca2+ release and give it more alkaline than ZOE (Tyagi et al., 2013). And due to the low cytotoxicity assessed by other studies, the CeraSeal RCS and BioRoo RCS inflammatory reaction reduces rapidly (Al‐Haddad et al., 2015), they have the potential to allow cell growth due to the release of calcium ions (Cosme‐Silva et al., 2019). Biocompatibility in vivo study showed that BioRoot RCS and CeraSeal had better biocompatibility than ZOE RCS for 21 days and there was a significant difference between study groups (0.000). The limitation of this study was firstly the disadvantages of a scanning electron microscope, starting with the size and cost. SEMs are expensive, large, and must be housed in an area free of any possible electric, magnetic, or vibration interference. So it will be necessary to use a modern device such as a confocal laser microscope for better results. For biocompatibility in the future, model studies can use contemporary sealer materials in the canal of the tooth of the dog for example will be required to enhance the rationale for the new materials and methodologies.

## 5 CONCLUSION

In conclusion, despite the limitations of this study, it can be concluded that both calcium silicate‐based sealers had sufficient sealing qualities and higher bond strength and biocompatibility than ZOE. The higher bond strength and biocompatibility of the two tested calcium silicate‐based sealers in conjunction with their bioactivity might help in the improvement of the root canal system treatment.

## AUTHOR CONTRIBUTIONS

All authors contributed to the manuscript revision. All authors approved the final version of the manuscript.

## CONFLICT OF INTEREST

The authors declare no conflicts of interest.

## Data Availability

The data that support the findings of this study are openly available in [repository name] at [URL], reference number [reference number].

## 1

See Tables A1 and A2, Figures A1, A2, A3, A4, A5, A6.

**Table A1:** ANOVA and LSD tests for mean inflammatory cells numbers of the four groups

Periods	Groups	*N*	Mean cells count	Standard error cells	Standard deviation cells	*p* Value	LSD (post hoc)	Conclusion
4 days	Bioroot	10	125.6	4.67	14.79	.647	‐	No significant differences
	CeraSeal	10	122.3	8.98	28.43		‐	
	ZOE	10	138.1	5.17	16.37		‐	
	Control	10	109.2	10.76	34.04		‐	
10 days	Bioroot	10	37.0	10.74	33.96	.000	‐	Significant differences
	CeraSeal	10	44.3	11.47	36.28		‐	
	ZOE	10	93.2	10.68	33.79		*	
	Control	10	43.4	11.99	37.94		‐	
21 days	Bioroot	10	4.2	1.22	3.88	.000	‐	Significant differences
	CeraSeal	10	4.9	1.15	3.64		‐	
	ZOE	10	17.6	1.82	5.78		*	
	Control	10	3.4	0.60	1.90		‐	

Abbreviations: ANOVA, analysis of variance; LSD, least significant difference.

**Table A2:** ANOVA and LSD tests for the tests RCS Groups

Groups	*N*	Min.	Max.	Mean	Standard deviation	Estimated *F*	*p* Value	LSD (post hoc)	Conclusion
Bioroot	30	0.00	1.60	0.485	0.346	5.978	.027	‐	Significant differences
CeraSeal	30	0.14	1.20	0.625	0.285			‐	
ZOE	30	2.00	4.20	3.242	0.579			*	

*Note*: *F*—Critical value with a degree of freedom (2, 27) = 3.354.

Abbreviations: ANOVA, analysis of variance; LSD, least significant difference; ZOE, Zinc oxide eugenol.

## References

[cre2652-bib-0001] Al‐Haddad, A. , Abu Kasim, N. H. , & Che Ab Aziz, Z. A. (2015). Interfacial adaptation and thickness of bioceramic‐based root canal sealers. Dental Materials Journal, 34(4), 516–521. 10.4012/dmj.2015-049 26235718

[cre2652-bib-0002] Bernath, M. , & Szabo, J. (2003). Tissue reaction initiated by different sealers. International Endodontic Journal, 36(4), 256–261. 10.1046/j.1365-2591.2003.00662.x 12702119

[cre2652-bib-0003] Camilleri, J. , Gandolfi, M. G. , Siboni, F. , & Prati, C. (2011). Dynamic sealing ability of MTA root canal sealer. International Endodontic Journal, 44(1), 9–21. 10.1111/j.1365-2591.2010.01774.x 20646079

[cre2652-bib-0004] Camps, J. , Jeanneau, C. , El Ayachi, I. , Laurent, P. , & About, I. (2015). Bioactivity of a calcium silicate‐based endodontic cement (BioRoot™ RCS): Interactions with human periodontal ligament cells in vitro. Journal of Endodontics, 41(9), 1469–1473. 26001857 10.1016/j.joen.2015.04.011

[cre2652-bib-0005] Cosme‐Silva, L. , Gomes‐Filho, J. E. , Benetti, F. , DalFabbro, R. , Sakai, V. T. , Cintra, L. T. , Ervolino, E. , & Viola, N. V. (2019). Biocompatibility and immunohistochemical evaluation of a new calcium silicate‐based cement, Bio‐C pulpo. International Endodontic Journal, 52(5), 689–700. 10.1111/iej.13052 30515845

[cre2652-bib-0006] Drukteinis, S. , Peciuliene, V. , Maneliene, R. , & Bendinskaite, R. S. (2009). In vitro study of microbial leakage in roots filled with EndoREZ sealer/EndoREZ Points and AH Plus sealer/conventional gutta‐percha points. Stomatologija, 11, 21–25. 19423967

[cre2652-bib-0007] El‐Mansy, L. H. , Mohamed Ali, M. , EL Sayed Hassan, R. , Ali Beshr, K. , & Hassan El Ashry, S. (2020). Evaluation of the biocompatibility of a recent bioceramic root canal sealer (BioRoot™ RCS): In‐vivo study. Journal of Medical Sciences, 8, 100–106.

[cre2652-bib-0008] Gambarini, G. , Seracchiani, M. , Zanza, A. , Miccoli, G. , Del Giudice, A. , & Testarelli, L. (2021). Influence of shaft length on torsional behavior of endodontic nickel–titanium instruments. Odontology, 109(3), 568–573. 10.1007/s10266-020-00572-2 33245455 PMC8178130

[cre2652-bib-0009] Gomes‐Filho, J. E. , Moreira, J. V. , Watanabe, S. , Lodi, C. S. , Cintra, L. T. , Dezan Junior, E. , Bernabé, P. F. , Nery, M. J. , & Otoboni Filho, J. A. (2012). Sealability of MTA and calcium hydroxide‐containing sealers. Journal of Applied Oral Science, 20(3), 347–351. 22858702 10.1590/S1678-77572012000300009 PMC3881769

[cre2652-bib-0010] Grossman, L. (1988). Endodontics (11th ed.). Lea & Febiger.

[cre2652-bib-0011] Hargreaves, K. M. , & Berman, L. H. (2016). Cohen's pathways of the pulp (11th ed.). Elsevier.

[cre2652-bib-0012] Holland, R. , Souza, V. , Nery, M. J. , Otoboni Filho, J. A. , Bernabé, P. F. E. , & Dezan Junior, E. (1999). Reactions of dogs' teeth to root canal filling with mineral trioxide aggregate or a glass ionomer sealer. Journal of Endodontics, 25, 728–730. 10726538 10.1016/s0099-2399(99)80118-6

[cre2652-bib-0013] Hovland, E. J. , & Dumsha, T. C. (1985). Leakage evaluation in vitro of the root canal sealer cement sealapex. International Endodontic Journal, 18, 179–182. 3861588 10.1111/j.1365-2591.1985.tb00437.x

[cre2652-bib-0014] Jain, P. R. , & Ranjan, M. A. (2015). The rise of biocramics in endodontics: A review. International Journal of Pharma and Bio Sciences, 61, 416–422.

[cre2652-bib-0015] Johnson, W. , Kulild, J. C. , & Tay, F. (2016). Obturation of the cleaned and shaped root canal system. In K. M. Hargreaves , & L. H. Berman (Eds.), Cohen's pathways of the pulp (pp. 280–322). Elsevier.

[cre2652-bib-0016] Khandelwal, D. , & Ballal, N. V. (2016). Recent advances in root canal sealers. International Journal of Clinical Dentistry, 9(3), 183–194.

[cre2652-bib-0017] Kharouf, N. , Arntz, Y. , Eid, A. , Zghal, J. , Sauro, S. , Haikel, Y. , & Mancino, D. (2020). Physicochemical and antibacterial properties of novel, premixed calcium silicate‐based sealer compared to powder–liquid bioceramic sealer. Journal of Clinical Medicine, 9(10), 3096. 32992852 10.3390/jcm9103096 PMC7600315

[cre2652-bib-0018] Kishen, A. , Peters, O. A. , Zehnder, M. , Diogenes, A. R. , & Nair, M. K. (2016). Advances in endodontics: Potential applications in clinical practice. Journal of Conservative Dentistry, 19(3), 199–206. 27217630 10.4103/0972-0707.181925 PMC4872571

[cre2652-bib-0019] López‐García, S. , Myong‐Hyun, B. , Lozano, A. , García‐Bernal, D. , Forner, L. , Llena, C. , Guerrero‐Gironés, J. , Murcia, L. , & Rodríguez‐Lozano, F. J. (2020). Cytocompatibility, bioactivity potential, and ion release of three premixed calcium silicate‐based sealers. Clinical Oral Investigations, 24, 1749–1759. 31399829 10.1007/s00784-019-03036-2

[cre2652-bib-0020] Mori, G. G. , Teixeira, L. M. , de Oliveira, D. L. , Jacomini, L. M. , & da Silva, S. R. (2014). Biocompatibility evaluation of biodentine in subcutaneous tissue of rats. Journal of Endodontics, 40(9), 1485–1488. 10.1016/j.joen.2014.02.027 25146039

[cre2652-bib-0021] Nair, U. , Ghattas, S. , Saber, M. , Natera, M. , Walker, C. , & Pileggi, R. (2011). A comparative evaluation of the sealing ability of 2 root‐end filling materials: An in vitro leakage study using *Enterococcus faecalis* . Oral Surgery, Oral Medicine, Oral Pathology, Oral Radiology, and Endodontics, 112(2), e74–e77. 21507688 10.1016/j.tripleo.2011.01.030

[cre2652-bib-0022] Peters, O. A. , Laib, A. , Rüegsegger, P. , & Barbakow, F. (2000). Three‐dimensional analysis of root canal geometry by high‐resolution computed tomography. Journal of Dental Research, 79(6), 1405–1409. 10890720 10.1177/00220345000790060901

[cre2652-bib-0023] Pinheiro, L. S. , Iglesias, J. E. , Boijink, D. , Mestieri, L. B. , Poli Kopper, P. M. , Figueiredo, J. A. P. , & Grecca, F. S. (2018). Cell viability and tissue reaction of NeoMTA plus: An in vitro and in vivo study. Journal of Endodontics, 44(7), 1140–1145. 10.1016/j.joen.2018.03.007 29866406

[cre2652-bib-0024] Ray, H. , & Seltzer, S. (1991). A new glass ionomer root canal sealer. Journal of Endodontics, 17(13), 598–603. 1820422 10.1016/s0099-2399(06)81832-7

[cre2652-bib-0025] Ricucci, D. , & Langeland, K. (1998). Apical limit of root canal instrumentation and obturation, part 2. A histological study. International Endodontic Journal, 31(6), 394–409. 10.1046/j.1365-2591.1998.00183.x 15551607

[cre2652-bib-0026] Santos, J. M. , Pereira, S. , Sequeira, D. B. , Messias, A. L. , Martins, J. B. , Cunha, H. , Palma, P. J. , & Santos, A. C. (2019). Biocompatibility of a bioceramic silicone‐based sealer in subcutaneous tissue. Journal of Oral Science, 61(1), 171–177. 10.2334/josnusd.18-0145 30918214

[cre2652-bib-0027] Simsek, N. , Akinci, L. , Gecor, O. , Alan, H. , Ahmetoglu, F. , & Taslidere, E. (2015). Biocompatibility of a new epoxy resin‐based root canal sealer in subcutaneous tissue of rat. European Journal of Dentistry, 9(1), 31–35. 10.4103/1305-7456.149635 25713481 PMC4319296

[cre2652-bib-0028] Trope, M. , Chow, E. , & Nissan, R. (1995). In vitro endotoxin penetration of coronally unsealed endodontically treated teeth. Endodontics and Dental Traumatology, 11(2), 90–94. 7641623 10.1111/j.1600-9657.1995.tb00465.x

[cre2652-bib-0029] Tyagi, S. , Mishra, P. , & Tyagi, P. (2013). Evolution of root canal sealers: An insight story. European Journal of General Dentistry, 2(3), 199–218.

[cre2652-bib-0030] Xuereb, M. , Vella, P. , Damidot, D. , Sammut, C. V. , & Camilleri, J. (2015). In situ assessment of the setting of tricalcium silicate‐based sealers using a dentin pressure model. Journal of Endodontics, 41(1), 111–124. 25442723 10.1016/j.joen.2014.09.015

